# Efficacy and Safety of Icotrokinra (an Interleukin-23 Inhibitor) in the Management of Moderate-to-Severe Plaque Psoriasis: A Systematic Review and Meta-Analysis

**DOI:** 10.31662/jmaj.2026-0001

**Published:** 2026-07-31

**Authors:** Zain Ahmad Siddiqi, Sume Rubab, Faisal Jamal Alashkar, Ayesha Iqbal, Niam Adam Abubakr Mahdi, Maheen Haque, Rutbah Amin Khairati, Tosin Ayantoyinbo, Kainat Qasim, Muhammad Ahmad Shahzaib, Anas Jamshed Khan, Fathima Masharifa, Muaz Shafique Ur Rehman, Fatimah Habib

**Affiliations:** 1 Department of Medicine, Allama Iqbal Medical College, Lahore, Pakistan; 2 Department of Dermatology, Mayo Hospital, Lahore, Pakistan; 3 Department of Medicine, Almaarefa University, Diriyah, Saudi Arabia; 4 Department of Medicine, Royal Shrewsbury Hospital, Shrewsbury, United Kingdom; 5 Department of Medicine, Hull University Teaching Hospitals NHS Trust, Hull, United Kingdom; 6 Department of Medicine, Dow International Medical College, Karachi, Pakistan; 7 Department of Internal Medicine, George Eliot Hospital, Nuneaton, United Kingdom; 8 Department of Internal Medicine, BronxCare Health System, New York, United States; 9 Department of Medicine, Al-Nafees Medical College and Hospital, Islamabad, Pakistan; 10 Department of Internal Medicine, Henan Medical University, Xinxiang, China; 11 Department of Anaesthesia, Buch International Hospital, Multan, Pakistan; 12 Department of Medicine, Sheikh Khalifa Medical City Hospital, Abu Dhabi, United Arab Emirates; 13 Department of Medicine, Jinnah Hospital, Lahore, Pakistan; 14 Department of Medicine, Scarborough General Hospital, Scarborough, United Kingdom

**Keywords:** plaque psoriasis, icotrokinra, interleukin-23, IL-23 inhibitor, Psoriasis Area and Severity Index, PASI-90, systematic review, meta-analysis

## Abstract

**Background:**

Plaque psoriasis, which is a chronic immune-mediated inflammatory skin condition, significantly reduces patients’ quality of life. The cytokine interleukin-23 (IL-23) is a pivotal factor in the development of psoriasis, and icotrokinra is a new oral IL-23 receptor antagonist that has shown potential effectiveness in clinical trials. The main goal of this systematic review and meta-analysis was to assess the effectiveness and safety of icotrokinra in adults and adolescents with moderate-to-severe plaque-type psoriasis.

**Methods:**

A comprehensive literature search in PubMed, ScienceDirect, and the Cochrane Library, conducted in accordance with Preferred Reporting Items for Systematic Reviews and Meta-Analyses (PRISMA) standards, identified randomized controlled trials that evaluated daily oral icotrokinra 200 mg versus placebo. The primary outcomes were Psoriasis Area and Severity Index (PASI) 90, Investigator’s Global Assessment (IGA) 0/1, and adverse events (AEs) at 16 weeks. Risk ratios with 95% confidence intervals were summed up through a random-effects model.

**Results:**

A total of five randomized controlled trials involving 2,755 participants were included. Icotrokinra showed a significant increase in PASI 90, IGA 0/1, IGA 0, and Psoriasis Symptoms and Signs Diary 0 responses compared to placebo. The overall frequency of AEs was similar in both groups, but the rate of treatment discontinuation due to AEs was lower with icotrokinra.

**Conclusions:**

Icotrokinra is an effective and well-tolerated oral treatment for moderate-to-severe plaque psoriasis with considerable clinical and patient-reported benefits.

## Introduction

Plaque psoriasis is an immune dysregulation–driven chronic inflammatory skin disease that presents as well-defined erythematous plaques with silvery scaling and affects a significant number of adults, with severe effects on quality of life ^(1), (2)^. The interleukin-23 (IL-23)/T helper 17 (Th17) pathway plays a central role in its pathogenesis, and targeted therapies against this axis have transformed the management of moderate-to-severe disease ^(3), (4)^.

The existing biologic therapies that target IL-23, including guselkumab, risankizumab, and tildrakizumab, work by binding to the p19 subunit of IL-23, thus hindering the IL-23/Th17 pathway, which results in considerable clinical responses as assessed by the Psoriasis Area and Severity Index (PASI) and Investigator’s Global Assessment (IGA) scores ^(5), (6)^. Based on meta-analyses, IL-23 inhibitors result in considerably higher PASI 75, PASI 90, and PASI 100 rates than placebo in moderate-to-severe plaque psoriasis, and safety is acceptable ^(7)^.

Although injectable IL-23 inhibitors have demonstrated strong efficacy in moderate-to-severe plaque psoriasis, the need for effective oral therapies persists to improve convenience, adherence, and accessibility across broader patient populations. Icotrokinra (JNJ-2113), a novel oral IL-23 receptor antagonist, has demonstrated promising efficacy and a favorable safety profile in recent clinical trials, including in adolescents and patients with difficult-to-treat disease sites ^(8), (9), (10)^. However, despite these encouraging findings, a comprehensive synthesis of the available evidence remains lacking.

Therefore, this study aimed to conduct a systematic review and meta-analysis of the efficacy and safety of icotrokinra in adolescents and adults with moderate-to-severe plaque psoriasis.

## Materials and Methods

We conducted this systematic review and meta-analysis in accordance with the established Preferred Reporting Items for Systematic Reviews and Meta-Analyses (PRISMA) guidelines after registering the protocol on PROSPERO (CRD420251267164) ^(11)^.

### Search strategy

PubMed, ScienceDirect, and the Cochrane Library were the three central databases used to systematically search for relevant articles using specific keywords combined with the OR and AND Boolean operators. The search strategy for ScienceDirect and PubMed was the same, but a research article filter was used on ScienceDirect. The search string used for both these databases was “(((Icotrokinra) OR (JNJ-77242113)) OR (IL-23 inhibitor)) AND (Plaque Psoriasis).” For the Cochrane Library, the search string used was “(Icotrokinra): ti, ab,kw OR (JNJ-77242113): ti, ab,kw OR (IL-23 inhibitor): ti, ab,kw AND (Plaque Psoriasis): ti, ab,kw.” No language restrictions were applied in the search. The studies were searched from inception until 31 December 2025.

### Selection criteria

We developed comprehensive eligibility criteria. Inclusion criteria were as follows: randomized controlled trials (RCTs) with participants aged 12 years or older with a confirmed diagnosis of psoriasis for a minimum duration of 6 months before the first dose of icotrokinra, with a PASI score >12 and an IGA score ≥3. The intervention was oral icotrokinra 200 mg. Outcomes were measured at 16 weeks and included PASI 90 (i.e., at least 90% improvement from baseline), IGA 0 or 1 (i.e., completely clear or almost clear skin), and adverse events (AEs) as the primary outcomes. Secondary outcomes included Psoriasis Symptoms and Signs Diary (PSSD) 0 and IGA 0. Articles reporting at least one primary outcome were included.

Articles were excluded if they were not RCTs, included participants younger than 12 years, or involved participants with a PASI score <12 or an IGA score <3 at baseline. Similarly, articles with pregnant or immunocompromised patients or patients with active tuberculosis were excluded. Articles that did not report at least one primary outcome were also excluded. Studies evaluating icotrokinra dosages other than 200 mg daily were removed as well.

### Quality assessment

We assessed the quality of the included studies using the revised Cochrane risk-of-bias tool for randomized trials (RoB 2) ^(12)^. RoB 2 comprises five domains, each evaluating a different aspect of trial quality, followed by an overall risk-of-bias assessment. Similarly, studies are considered at low risk of bias if all domains are rated as low risk. An overall rating of some concerns is assigned if one domain raises some concerns while the remaining domains are rated as low risk.

### Screening, selection, and data extraction

Two reviewers independently screened and selected articles, with any disputes resolved by a third reviewer. Initially, we screened for duplicates, after which the remaining articles underwent title screening, followed by abstract screening in case of any uncertainty. Full texts of potentially eligible articles were then assessed against the eligibility criteria. Articles that fulfilled our criteria were included in the final analysis.

Data from the included articles were extracted by one reviewer and reviewed by another author. The following data were extracted into an Excel spreadsheet (Microsoft Corporation, Redmond, WA, USA): primary author, trial identifier, study year, sample size, age, male-to-female ratio, baseline PASI and IGA scores, patient characteristics, intervention used, and outcome data.

### Data synthesis

The statistical software RevMan 5.4 (The Cochrane Collaboration, Copenhagen, Denmark) was used to analyze the data from the included articles ^(13)^. For dichotomous data, risk ratios (RRs) were used as the outcome measure. The Mantel-Haenszel model was applied with random effects and 95% confidence intervals (CIs). Statistical significance was established at the 0.05 level. Forest plots were generated to visually present the data, and a sensitivity analysis was conducted to assess heterogeneity and increase the robustness of our findings. A leave-one-out sensitivity analysis was performed, and the results were rechecked for any significant differences. Heterogeneity was classified as low for I^2^ <25%, mild for I^2^ = 26%-50%, moderate for I^2^ = 51%-75%, and high for I^2^ >75%.

## Results

Initially, 2,740 articles were retrieved from the three selected databases: 347 from PubMed, 2,256 from ScienceDirect, and 137 from the Cochrane Library. After duplicate screening, 57 articles were removed. A total of 2,672 articles were removed during title and abstract screening, which was performed independently by two reviewers, and the full texts of 11 articles were assessed for eligibility. Of these 11 articles, only 5 fulfilled our selection criteria and were included in our final analysis. The article by Gold et al. ^(14)^ reported on both the ICONIC-ADVANCE 1 and ICONIC-ADVANCE 2 trials. The selection process is summarized in Figure 1.

**Figure 1.**
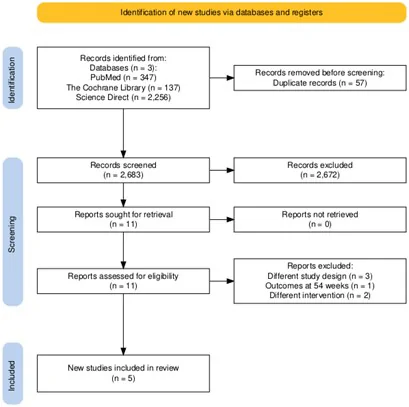
PRISMA flowchart for article screening and selection. PRISMA: Preferred Reporting Items for Systematic Reviews and Meta-Analyses.

### Characteristics of studies

Our systematic review comprised five RCTs with distinct trial identifiers, involving 2,755 participants. The majority of participants were male (n = 1,847), whereas 908 were female. Four studies reported baseline PASI scores as means, whereas one reported the score as a median (interquartile range). The icotrokinra dose was consistent across all trials at 200 mg daily. A total of 1,339 participants were assigned to the experimental group and 612 to the control group. At baseline, 2,144 participants had moderate plaque psoriasis, whereas 594 had severe plaque psoriasis. All trials reported on our selected outcomes except the study by Gooderham et al. ^(15)^, in which the PSSD score was not reported. Key characteristics of the included studies are presented in Table 1^(14), (15), (16), (17)^.

**Table 1.** Characteristics of Included Studies.

Study	Trial identifier	Sample size (n)	Age (years), mean (SD)	Male:female	Intervention (n)	Comparator (n)	Baseline PASI	Baseline IGA (n)		Outcomes
								Moderate	Severe	
Bissonnette et al. ^(16)^, 2024	NCT05223868	255	44.3 (12.65)	176:79	Icotrokinra 200 mg per oral daily (42)	Placebo (43)	Mean (SD): 19.05 (5.83)	202	53	PASI scores, IGA, PSSD score, DLQI score, and adverse events
Bissonnette et al. ^(17)^, 2025	NCT06095115	684	42.6 (16.4)	447:237	Icotrokinra 200 mg per oral daily (456)	Placebo (228)	Mean (SD): 19.9 (7.5)	514	170	PASI scores, IGA, PSSD score, and adverse events
Gold et al. ^(14)^, 2025 (ADVANCE 1)	NCT06143878	774	46.7 (13.4)	528:246	Icotrokinra 200 mg per oral daily (311)	Placebo (156)	Median (IQR): 18.3 (15.2–22.9)	616	158	PASI scores, IGA, PSSD score, and adverse events
Gold et al. ^(14)^, 2025 (ADVANCE 2)	NCT06220604	731	46.1 (13.6)	496:235	Icotrokinra 200 mg per oral daily (322)	Placebo (82)	Median (IQR): 17.9 (15.2–22.0)	586	145	PASI scores, IGA, PSSD score, and adverse events
Gooderham et al. ^(15)^, 2025	NCT06095102	311	44.7 (14.3)	200:111	Icotrokinra 200 mg per oral daily (208)	Placebo (103)	Mean (SD): 16.3 (12.5)	226	68	PSSD score, IGA, and adverse events

DLQI: Dermatology Life Quality Index; IGA: Investigator’s Global Assessment; IQR: interquartile range; PASI: Psoriasis Area and Severity Index; PSSD: Psoriasis Symptoms and Signs Diary; SD: standard deviation.

### Quality assessment

Risk of bias assessed using RoB 2 showed low risk across all domains in all trials, indicating an overall low risk of bias and suggesting that the included studies were of high quality. Figure 2 presents a summary of the quality assessment.

**Figure 2.**
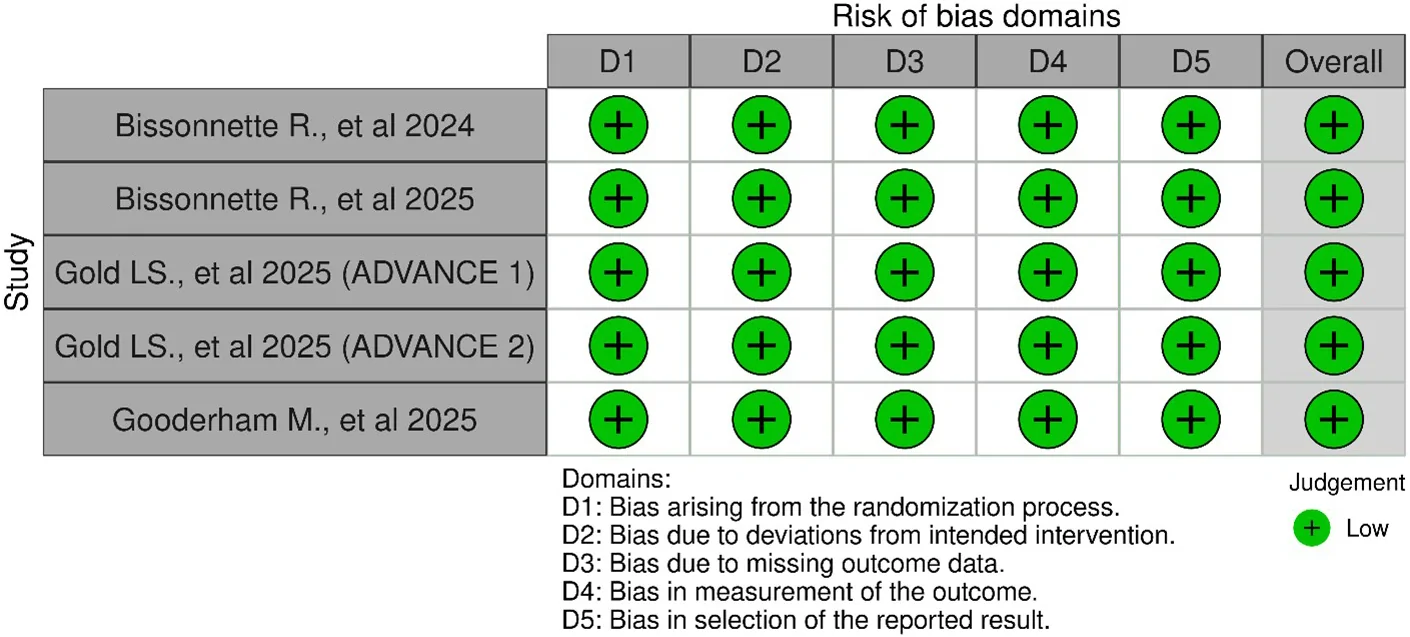
Summary of risk of bias.

### IGA category

All included studies reported on both IGA 0/1 and IGA 0 outcomes at 16 weeks. For IGA 0/1, the analysis showed significantly greater improvement with icotrokinra than with placebo; however, moderate statistical heterogeneity was observed, that is, I^2^ = 51% (RR 6.45, 95% CI 4.47-9.33; p < 0.00001). Sensitivity analysis showed that, despite no change in results, heterogeneity was reduced to 0% after removal of the study by Bissonnette et al. ^(16)^ (RR 7.47, 95% CI 5.70-9.79; p < 0.00001).

Similarly, for IGA 0, the results showed that icotrokinra was significantly associated with clearer skin compared to placebo at 16 weeks (RR 24.11, 95% CI 12.31-47.20; p < 0.00001), with no observed heterogeneity. A forest plot for both results is presented in Figure 3.

**Figure 3.**
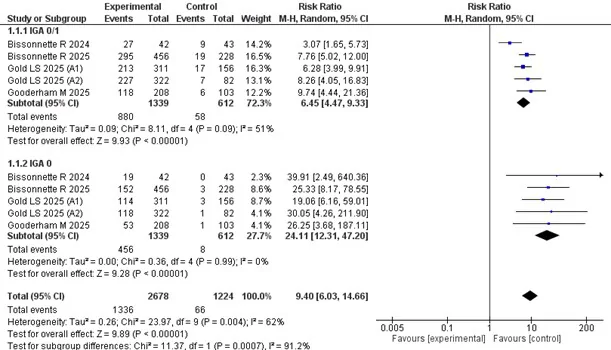
Forest plot showing results for IGA improvement between icotrokinra and placebo. IGA: Investigator’s Global Assessment.

### PASI 90

The analysis of a 90% improvement in the PASI showed significant results in favor of icotrokinra compared to placebo at 16 weeks. More participants in the intervention group achieved PASI 90, with no statistical heterogeneity (RR 13.82, 95% CI 8.75-21.84; p < 0.00001). The forest plot in Figure 4 summarizes the PASI 90 results.

**Figure 4.**
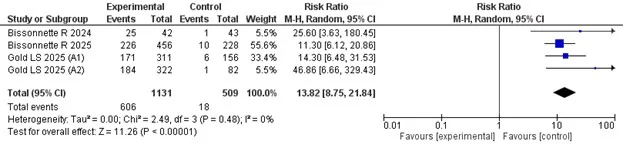
Forest plot summarizing the PASI 90 results. PASI: Psoriasis Area and Severity Index.

### PSSD 0

Similar to the favorable results observed for PASI 90 and IGA scores, icotrokinra presented more favorable and significant results with more participants achieving a PSSD score of 0 compared to placebo, with low heterogeneity, that is, I^2^ = 4% (RR 9.72, 95% CI 5.08-18.59; p < 0.00001). The forest plot in Figure 5 presents the results.

**Figure 5.**
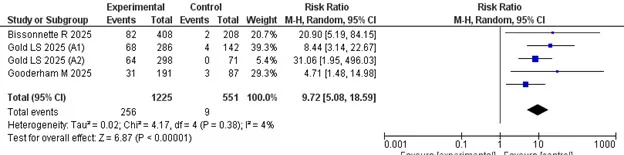
Forest plot of PSSD 0 outcomes. PSSD: Psoriasis Symptoms and Signs Diary.

### AEs

We evaluated any AE, serious AEs, and AEs that led to treatment discontinuation. The most frequently reported AEs across all trials were headache, upper respiratory tract infection, and nasopharyngitis. For any AE, no significant difference was observed between the icotrokinra and placebo groups (RR 1.07, 95% CI 0.94-1.22; p = 0.30), with no heterogeneity, that is, I^2^ = 0%. For serious AEs, the risk was significantly decreased with icotrokinra compared to placebo (RR 0.25, 95% CI 0.09-0.67; p = 0.006), with no heterogeneity, that is, I^2^ = 0%.

Similarly, the risk of treatment discontinuation due to AEs was lower with icotrokinra than with placebo but without any significance and with no heterogeneity (RR 0.86, 95% CI 0.26-2.89; p = 0.81). The forest plot of AEs is presented in Figure 6.

**Figure 6.**
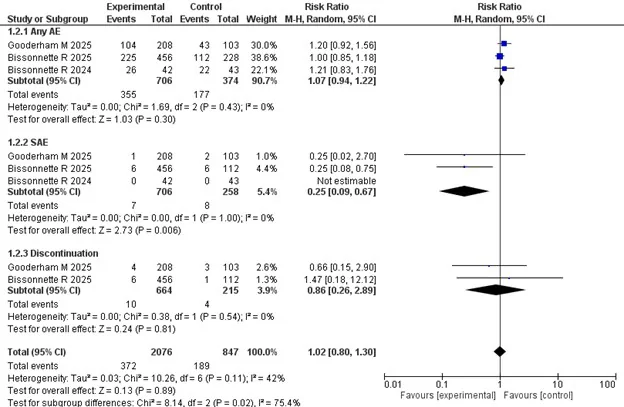
Forest plot of any AE, serious AEs, and AEs that led to treatment discontinuation. AE: adverse event.

## Discussion

This systematic review and meta-analysis provides strong evidence for the use of oral icotrokinra as a highly effective treatment for moderate-to-severe plaque psoriasis, resulting in significant enhancements in both observer-reported and patient-reported outcomes at 16 weeks. Across five RCTs involving more than 2,700 participants, icotrokinra showed considerable superiority compared to placebo in reaching PASI 90, IGA 0/1, IGA 0, and PSSD-0, while also demonstrating a positive safety and tolerability profile.

The methodological quality of the included studies further strengthens these findings. All trials were assessed using RoB 2 and demonstrated low risk of bias across all domains, reflecting high-quality evidence. This is consistent with methodological guidance suggesting that the standardized application of RoB 2 improves the reliability of bias assessment in randomized trials ^(18)^. Sensitivity analyses further supported the robustness of the results. Exclusion of the study by Bissonnette et al. ^(16)^ reduced heterogeneity because of its phase 2 dose-finding design, inclusion of multiple dosing regimens, and differences in baseline disease severity and outcome measures compared to the phase 3 trials. Similarly, the ADVANCE trials contributed to heterogeneity because of their large multicenter design, more diverse patient populations, and variations in study structure and AE reporting. Removal of these studies eliminated heterogeneity without affecting the overall effect estimates.

The observed changes in IGA and PASI outcomes are in line with phase 3 trials, such as ICONIC-LEAD and ICONIC-ADVANCE 1 and 2, which also reported high response rates with icotrokinra ^(14), (19)^. This consistency across trials strengthens the reliability of the results.

Beyond the clinician-assessed outcomes, treatment with icotrokinra was associated with a marked increase in the number of patients who reached PSSD 0, indicating complete resolution of psoriasis-related symptoms. This result is in line with earlier validation studies demonstrating that the PSSD is a trustworthy patient-reported outcome measure, with high correlations with PASI and IGA scores ^(20)^. The increase in PSSD 0 responses emphasizes that the clinical benefits of icotrokinra translate into significant symptom reduction from the patient’s point of view.

There were also safety findings consistent with the current evidence. There was no significant difference in the incidence of overall and serious AEs between icotrokinra and placebo, and the incidence of treatment discontinuation due to AEs was lower with icotrokinra. The majority of AEs reported were mild, supporting the favorable tolerability profile of icotrokinra and aligning with the results of major clinical trials ^(21)^.

Overall, these findings suggest that icotrokinra represents an effective and well-tolerated oral therapeutic option for moderate-to-severe plaque psoriasis, offering a convenient alternative to injectable biologic therapies.

### Limitations

It should be noted that this study has several limitations. First, the maximum follow-up duration across the included trials was only 16 weeks, which limited the evaluation of long-term effectiveness, response persistence, and overall treatment safety. Second, icotrokinra was primarily compared with placebo in the included studies, precluding extensive comparisons with established biologics or other oral agents. Third, although mean heterogeneity was low across the board, IGA 0/1 outcomes showed moderate heterogeneity, partly because of differences in study design and population characteristics. Additionally, major databases such as Embase and Web of Science were not searched because of limited access, which could have resulted in omission of relevant articles. Similarly, RoB 2 was not used to assess specific outcomes, which could have introduced bias into the results. Furthermore, ADVANCE trials could not be included in the safety analysis because separate safety data were unavailable.

Additionally, only a single predetermined dose of icotrokinra (200 mg once daily) was administered, precluding dose-response analyses. Finally, trial participants may not fully represent the variation of patients seen in the clinic, such as those with multiple comorbidities or prior biologic treatment failure, which could limit the applicability of the findings.

### Future directions

Long-term extension studies are needed to evaluate the sustained efficacy, relapse rates after treatment discontinuation, and long-term safety of icotrokinra. Head-to-head trials of icotrokinra versus established biologics and other oral targeted therapies would provide valuable insights into its relative effectiveness and potential role in treatment pathways. In addition, real-world evidence studies are required to examine treatment adherence, patient preference, and outcomes in more extensive and diverse populations. Moreover, investigating icotrokinra’s use in exceptional populations, including patients previously treated with biologics and those with psoriatic arthritis or other inflammatory comorbidities, may further clarify its clinical value.

### Conclusion

This systematic review and meta-analysis provides evidence that icotrokinra is a safe and effective oral medication for patients with moderate-to-severe plaque psoriasis. The drug significantly outperforms placebo in both skin clearance and patient-reported outcomes, and its safety profile is on par with placebo. As the first oral IL-23 receptor antagonist, icotrokinra is a significant step forward in the treatment of psoriasis and offers a good alternative to injectable biologic therapies. However, more long-term and comparative studies are needed to further establish its role in clinical practice.

## Article Information

### Acknowledgments

Rutbah Amin Khairati (7th author) contributed equally to this work and should be recognized as a co-first (joint first) author.

### Author Contributions

Zain Ahmad Siddiqi: conceptualization, study design, and overall supervision of the project. Sume Rubab: clinical input, dermatological assessment, and contribution to patient-related data interpretation. Faisal Jamal Alashkar: data collection, methodological assistance, and review of internal medicine–related outcomes. Ayesha Iqbal: clinical evaluation, patient management input, and critical review of the manuscript. Niam Adam Abubakr Mahdi: data curation and assistance in drafting the initial version of the manuscript. Maheen Haque: statistical support, data validation, and contribution to results interpretation. Rutbah Amin Khairati: literature review, data acquisition, and contribution to the discussion section. Tosin Ayantoyinbo: clinical relevance assessment, manuscript editing, and quality assurance. Kainat Qasim: referencing, formatting, and manuscript organization. Muhammad Ahmad Shahzaib: data acquisition and contribution to methodology and results sections. Anas Jamshed Khan: intensive care unit/anesthesia–related clinical input and critical appraisal of patient safety aspects. Fathima Masharifa: contribution to data collection and assistance in manuscript drafting. Muaz Shafique Ur Rehman: review of clinical implications, proofreading, and final content validation. Fatimah Habib: project coordination, manuscript refinement, communication with the journal, and final approval of the version to be published. All authors approved the final version of the manuscript and agree to be accountable for all aspects of the work.

### Conflicts of Interest

None

### Ethical Considerations

Ethics approval was not required as this study is a systematic review and meta-analysis of previously published data.

## Supplement

Supplementary Material
