# Supplementary material for: Efficacy and Safety of Icotrokinra (an Interleukin-23 Inhibitor) in the Management of Moderate-to-Severe Plaque Psoriasis: A Systematic Review and Meta-Analysis
Source: JMA J. 2026 Jul 31;9(5):1397–405. doi: 10.31662/jmaj.2026-0001 (PMC13639414; doi:10.31662/jmaj.2026-0001)
Supplement: Supplementary file 1 [file 2433-3298-9-5-1397-s001.pdf]

Pubmed

(((Icotrokinra) OR (JNJ-77242113)) OR (IL-23 inhibitor)) AND (Plaque Psoriasis)

Science direct

(((Icotrokinra) OR (JNJ-77242113)) OR (IL-23 inhibitor)) AND (Plaque Psoriasis)

Filter: research article

Cochrane

(Icotrokinra): ti, ab,kw OR (JNJ-77242113): ti, ab,kw OR (IL-23 inhibitor): ti, ab,kw AND (Plaque Psoriasis): ti, ab,kw'
